# Temporal trends and patterns in heart failure incidence: a population-based study of 4 million individuals

**DOI:** 10.1016/S0140-6736(17)32520-5

**Published:** 2018-02-10

**Authors:** Nathalie Conrad, Andrew Judge, Jenny Tran, Hamid Mohseni, Deborah Hedgecott, Abel Perez Crespillo, Moira Allison, Harry Hemingway, John G Cleland, John J V McMurray, Kazem Rahimi

**Affiliations:** a The George Institute for Global Health, University of Oxford, Oxford, UK; b Deep Medicine, Oxford Martin School, University of Oxford, Oxford, UK; c Nuffield Department of Orthopaedics, Rheumatology and Musculoskeletal Sciences, Nuffield Orthopaedic Centre, University of Oxford, Oxford, UK; d Bristol National Institute for Health Research Biomedical Research Centre, Musculoskeletal Research Unit, Southmead Hospital, University of Bristol, Bristol, UK; e Medical Research Council Lifecourse Epidemiology Unit, Southampton General Hospital, University of Southampton, Southampton, UK; f The Farr Institute of Health Informatics Research, University College London, London, UK; g The National Institute for Health Research, Biomedical Research Centre, University College London Hospitals NHS Foundation Trust/University College London, London, UK; h Robertson Centre for Biostatistics and Clinical Trials, University of Glasgow, Glasgow, UK; i Institute for Cardiovascular and Medical Sciences, University of Glasgow, Glasgow, UK; j National Heart & Lung Institute, Imperial College London, London, UK; k Oxford University Hospitals NHS Foundation Trust, Oxford, UK

## Abstract

**Background:**

Large-scale and contemporary population-based studies of heart failure incidence are needed to inform resource planning and research prioritisation but current evidence is scarce. We aimed to assess temporal trends in incidence and prevalence of heart failure in a large general population cohort from the UK, between 2002 and 2014.

**Methods:**

For this population-based study, we used linked primary and secondary electronic health records of 4 million individuals from the Clinical Practice Research Datalink (CPRD), a cohort that is representative of the UK population in terms of age and sex. Eligible patients were aged 16 years and older, had contributed data between Jan 1, 2002, and Dec 31, 2014, had an acceptable record according to CPRD quality control, were approved for CPRD and Hospital Episodes Statistics linkage, and were registered with their general practice for at least 12 months. For patients with incident heart failure, we extracted the most recent measurement of baseline characteristics (within 2 years of diagnosis) from electronic health records, as well as information about comorbidities, socioeconomic status, ethnicity, and region. We calculated standardised rates by applying direct age and sex standardisation to the 2013 European Standard Population, and we inferred crude rates by applying year-specific, age-specific, and sex-specific incidence to UK census mid-year population estimates. We assumed no heart failure for patients aged 15 years or younger and report total incidence and prevalence for all ages (>0 years).

**Findings:**

From 2002 to 2014, heart failure incidence (standardised by age and sex) decreased, similarly for men and women, by 7% (from 358 to 332 per 100 000 person-years; adjusted incidence ratio 0·93, 95% CI 0·91–0·94). However, the estimated absolute number of individuals with newly diagnosed heart failure in the UK increased by 12% (from 170 727 in 2002 to 190 798 in 2014), largely due to an increase in population size and age. The estimated absolute number of prevalent heart failure cases in the UK increased even more, by 23% (from 750 127 to 920 616). Over the study period, patient age and multi-morbidity at first presentation of heart failure increased (mean age 76·5 years [SD 12·0] to 77·0 years [12·9], adjusted difference 0·79 years, 95% CI 0·37–1·20; mean number of comorbidities 3·4 [SD 1·9] *vs* 5·4 [2·5]; adjusted difference 2·0, 95% CI 1·9–2·1). Socioeconomically deprived individuals were more likely to develop heart failure than were affluent individuals (incidence rate ratio 1·61, 95% CI 1·58–1·64), and did so earlier in life than those from the most affluent group (adjusted difference −3·51 years, 95% CI −3·77 to −3·25). From 2002 to 2014, the socioeconomic gradient in age at first presentation with heart failure widened. Socioeconomically deprived individuals also had more comorbidities, despite their younger age.

**Interpretation:**

Despite a moderate decline in standardised incidence of heart failure, the burden of heart failure in the UK is increasing, and is now similar to the four most common causes of cancer combined. The observed socioeconomic disparities in disease incidence and age at onset within the same nation point to a potentially preventable nature of heart failure that still needs to be tackled.

**Funding:**

British Heart Foundation and National Institute for Health Research.

## Introduction

Adequate public health and service delivery planning requires reliable information about contemporary population-level disease incidence. Policies need to consider both standardised rates, which describe disease incidence independently of changes in population, and absolute numbers of patients affected, which describe the impact of the disease on the population and services of interest.

Estimates of heart failure incidence and its temporal trends, even in high-income countries, are scarce and inconsistent.^1^, ^2^, ^3^, ^4^, ^5^, ^6^ Previous studies have frequently been based on selected cohorts,^2^, ^3^, ^4^, ^6^ which may not represent the general population. Other studies have restricted case identification to those made in general practice consultations^7^ or hospital admissions.^8^, ^9^ However, it is only by considering presentations across the whole spectrum of acute and chronic care that the full burden of disease can be captured and an accurate distinction made between incident and prevalent cases. Moreover, previous reports have differed in their approach to age standardisation—with some authors presenting crude rates,^5^, ^10^ others standardising to census populations,^3^, ^4^, ^6^ or simply adjusting for differences in the denominator population.^2^, ^11^ Very few studies refer to a standard population, rendering comparisons between studies challenging. Consequently, reported incidence varies by a factor of ten across studies (appendix).

**Research in context**

**Evidence before this study**

We searched PubMed for reports published in English between Jan 1, 2000, and April 30, 2017, that included “heart failure” and “incidence” in their title. We also reviewed references of clinical practice guidelines and consulted with experts for relevant studies.

Estimates of heart failure incidence rates, trends over time, and association with patient features were scarce and inconsistent. Studies frequently referred to restricted populations or to limited data sources, from either general practice consultations or hospital admissions. To compare reports of heart failure incidence, we selected a subset of studies that referred to a community-based cohort, included outpatient diagnoses in their case identification, presented incident rates in the general population (all ages and sexes), and considered all types of heart failure. We found that reports differed in their approach to age-standardisation, rendering comparisons between studies challenging, and that reported incidence rates varied by a factor of ten across studies. We found no study that presented both crude and standardised rates overall and by subgroups.

**Added value of this study**

Our study provides important new information on the contemporary incidence of heart failure and insights into its variation over time by age, sex, region, and socioeconomic status. We present crude and standardised incidence rates derived from a large, representative, general population cohort, setting a baseline for international comparison, monitoring of prevention strategies, and informed design of public health policies. We provide evidence that, in the UK, the burden of heart failure is increasing with population growth and ageing, and is similar to the combined incidence of breast, prostate, lung, and bowel cancer. Many high-income countries have similar population structures and temporal changes, and are likely to have a similar burden. Our results also highlight important changes in patients' profile over time—with a trend towards an increasing age at first presentation and a substantial rise in the number of associated comorbidities—and quantifies the magnitude of socioeconomic disparities, showing that these affect not only incidence of heart failure, but also age at first presentation and prevalence of comorbidities.

**Implications of all the available evidence**

In Europe, North America, and Australasia, improvements in heart failure prevention have been achieved but remain modest compared with other cardiovascular diseases. Population growth and ageing, observed in many high-income countries, have been shown to overcome these improvements, leading to an increasing absolute burden of heart failure. Incidence of heart failure is similar to the four most common causes of cancer (lung, breast, bowel, and prostate) combined, and requires reinforced public health action in prevention and resource planning, as well as efficient and effective care delivery. The profile of patients with heart failure, although mostly elderly, male, multimorbid, and economically deprived individuals, remains diverse and is changing over time. Socioeconomic disparities in disease incidence, age at onset, as well as associated comorbidities point to a potentially preventable nature of heart failure that still needs to be tackled.

As for the absolute number of cases of incident heart failure, country-specific estimates are rarely reported. For instance, in the UK, although several national programmes generate heart failure statistics, reliable information about the absolute number of new cases is not available. The Annual Quality and Outcomes Framework reports UK prevalence estimates based on general practice consultations, but yearly incidence is not recorded.^12^ The British Heart Foundation's Cardiovascular Disease Statistics and the National Heart Failure Audit include annual numbers of inpatient episodes of heart failure, but are limited in their ability to distinguish between first and recurrent presentations.^13^, ^14^ More generally, both national and international quantification of the absolute number of cases of incident heart failure are absent from global heart failure reviews.^15^, ^16^ Additionally, long-term trends on the heterogeneity of incident heart failure in the general population overall, and by features such as age or socioeconomic status, are scarce.^17^

To address these knowledge gaps, we used a large longitudinal database of linked primary and secondary care records from a representative sample of the UK population^18^ to assess trends in crude and standardised heart failure incidence by sex, age, socioeconomic status and region. We also investigated the comorbidity profile of patients over more than a decade.

## Methods

### Data sources

We used electronic health records from the Clinical Practice Research Datalink (CPRD) from Jan 1, 1985, to Sept 30, 2015. The CPRD database contains anonymised patient data from approximately 7% of the UK population and is broadly representative in terms of age, sex, and ethnicity. CPRD is one of the largest databases of longitudinal medical records from primary care in the world and has been validated for epidemiological research for a broad range of conditions.^18^ Primary care records from CPRD were linked to secondary care admission records from Hospital Episodes Statistics Admitted Patient Care data. Linkage was available for a subset of English practices from Jan 1, 1998, covering approximately 50% of all CPRD records. Scientific approval for this study was given by the CPRD Independent Scientific Advisory Committee (ISAC).

### Study population

Patients were men and women aged 16 years and older, contributing to data between Jan 1, 2002, and Dec 31, 2014. Patients were eligible for inclusion if their record was labelled as acceptable by CPRD quality control^18^ and approved for CPRD and Hospital Episodes Statistics linkage, and if the patient was registered with their general practice for at least 12 months.

For incidence calculations, we excluded all individuals who had a diagnosis of heart failure before the study start date (Jan 1, 1985, to Jan 1, 2002, in primary care records and Jan 1, 1998, to Jan 1, 2002, in secondary care records), or within the first 12 months of registration with their general practice.

### Case identification and categorisation

To identify heart failure diagnoses, we used a list of 107 diagnostic codes from hospital (International Classification of Diseases, tenth revision [ICD-10]) and primary care (Read^19^) coding schemes (appendix). These include all codes used in the UK quality of care management programmes (the Annual Quality and Outcomes Framework introduced in 2004 for primary care, and the National Heart Failure Audit introduced in 2007 for secondary care), and additional codes identified through medical dictionary keyword searches, previously published literature,^4^, ^7^ and online clinical code repositories.^20^ We defined incident heart failure diagnosis as the first record of heart failure in primary care or hospital admission records from any diagnostic position.

### Patient characteristics

For patients with incident heart failure, we extracted the most recent measurement of baseline characteristics within 2 years of a diagnosis of heart failure—ie, systolic and diastolic blood pressure, smoking status, and body-mass index (BMI)—from electronic health records. BMI was categorised as underweight (<18·5 kg/m^2^), normal (18·5–24·9 kg/m^2^), overweight (25–29·9 kg/m^2^), and obese (≥30 kg/m^2^).

We also extracted information about comorbidities, socioeconomic status, ethnicity, and geographical region.

To describe comorbidities, we selected 17 common chronic conditions: anaemia, asthma, atrial fibrillation, cancer, chronic kidney disease, chronic obstructive pulmonary disease, dementia, depression, diabetes, dyslipidaemia, hypertension, ischaemic heart disease, obesity, osteoarthritis, peripheral arterial disease, stroke, and thyroid disease. For each condition, we report prevalence as the percentage of patients with a diagnosis recorded in their primary care or hospital discharge record, before their first diagnosis of heart failure. Patients without diagnosis were assumed to be free from that condition. Diagnosis code lists for the extraction of each condition were adapted from the CALIBER code repository.

To describe socioeconomic status, we used the Index of Multiple Deprivation (IMD) 2015 quintile,^21^ a composite measure of relative deprivation at a small area level, covering an average population of 1500 people, ranked in ascending order of deprivation score and grouped in equal fifths, with quintile 1 representing the least deprived area and quintile 5 representing the most deprived area (appendix). Although absence of deprivation does not necessarily equate to affluence, to assist readability we refer to least deprived and most affluent interchangeably. Similarly, although the IMD quintiles refer to small neighbourhoods rather than to individuals, we use the terms most deprived or affluent to refer to people living in the most deprived or affluent areas.

### Statistical analyses

Baseline characteristics are presented as frequencies (%) for categorical data, medians and IQR for non-normally distributed continuous data, or means and SD for normally distributed continuous data. Data are stratified by sex, socioeconomic quintile, and period of diagnosis. Number and percentage of records with missing data are displayed for variables with missing entries. For categorical variables, frequencies refer to complete cases.

We computed age-specific, sex-specific, and year-specific incidence by dividing the number of incident cases by the number of patient-years in the cohort. Time at risk was restricted to number of days alive, in people aged 16 years and older, who were registered with a general practice for over 12 months, and to practice's up-to-standard periods. We calculated age-specific, sex-specific, and year-specific prevalence considering all patients ever diagnosed with heart failure (numerator) among patients alive, aged 16 years or older, and registered with a general practitioner during an up-to-standard period (denominator) on June 30 in each year. We assumed no heart failure for patients aged 15 years or younger and report total incidence and prevalence for all ages (>0 years).

To calculate standardised rates, we applied direct age and sex standardisation^22^ to the 2013 European Standard Population^23^ using 5-year age bands up to 90 years of age. The European Standard Population is an artificial population structure, designed and published by the statistical office of the European Union (Eurostat), to allow the calculation of age-standardised and sex-standardised rates that are comparable across regions and time.^23^

Crude rates were inferred by applying year-specific, age-specific, and sex-specific incidence to UK census mid-year population estimates, using 5-year age bands up to 90 years of age.

We used Poisson regression models to examine overall and category-specific incidence ratios and corresponding 95% CIs. When applicable, we adjusted models for time, age, sex, region, and socioeconomic status.

Study findings are reported in accordance with the REporting of studies Conducted using Observational Routinely-collected health Data (RECORD) recommendations.^24^ We used R (version 3.3) to do statistical analyses.

### Role of the funding source

The funders of the study had no role in study design, data collection, data analysis, data interpretation, or writing of the report. NC and KR had full access to the data in the study and had final responsibility for the decision to submit for publication.

## Results

A total of 4 045 144 patients contributed data between Jan 1, 2002, and Dec 31, 2014. For incidence calculations, we excluded all individuals who had a diagnosis of heart failure before the study start date (45 671 records) or within the first 12 months of registration with their general practice (7056 records), leading to 3 992 417 eligible patients, and a total of 24 877 519 patient-years at risk. Of those patients, 93 074 developed incident heart failure during the study period. Patient characteristics stratified by sex, socioeconomic status, and time period categories are shown in the table. Mean age at heart failure diagnosis was 76·7 years (SD 12·6), and 49·0% were women. From 2002 to 2014, mean age at diagnosis increased from 76·5 (12·0) to 77·0 years (12·9; adjusted difference 0·79 years, 95% CI 0·37–1·20; table). Men had higher prevalence of smoking than did women, and individuals who were most deprived had a higher prevalence of smoking and were more likely to have a body-mass index (BMI) in the overweight or obese range at baseline than the most affluent individuals (table).

**Table:** Characteristics of patients with incident heart failure

		**All patients (n=93 074)**	**Sex**		**Socioeconomic status**		**Time period**	
			Female (n=45 647)	Male (n=47 427)	SES 1 (n=18 371)	SES 5 (n=16 270)	2002–04 (n=21 943)	2012–14 (n=20 804)
Age (years)		76·7 (12·6)	79·4 (11·8)	74·0 (12·7)	77·8 (12·1)	74·5 (13·3)	76·5 (12·0)	77·0 (12·9)
	Sex							
	Women	45 647 (49%)	··	··	8694 (48%)	8278 (52%)	10 889 (50%)	10 163 (50%)
	Men	47 427 (51%)	··	··	9510 (52%)	7612 (48%)	10874 (50%)	10106 (50%)
Ethnicity*								
	White	45 550 (97%)	22 247 (98%)	23 303 (97%)	9108 (98%)	8330 (96%)	10 588 (98%)	13 618 (96%)
	Missing data	46 278 (50%)	22 875 (50%)	23 403 (49%)	9096 (50%)	7560 (46%)	11 175 (51%)	6651 (32%)
Socioeconomic status quintile								
	Quintile 1 (least deprived)	18 371 (20%)	8694 (19%)	9677 (20%)	··	··	4177 (19%)	4344 (21%)
	Quintile 2	20 073 (22%)	9737 (21%)	10 336 (22%)	··	··	4680 (21%)	4600 (22%)
	Quintile 3	20 052 (22%)	9818 (21%)	10 234 (22%)	··	··	4769 (22%)	4500 (22%)
	Quintile 4	18 308 (20%)	9120 (20%)	9188 (19%)	··	··	4387 (20%)	3941 (19%)
	Quintile 5 (most deprived)	16 270 (17%)	8278 (18%)	7992 (17%)	··	··	3930 (18%)	3419 (16%)
Systolic blood pressure								
	Mean (mm Hg)	133 (21)	134 (21)	131 (21)	132 (20)	132 (21)	137 (24)	130 (19)
	Missing data	5195 (6%)	2716 (6%)	2479 (5%)	922 (5%)	1057 (6%)	2601 (12%)	645 (3%)
Diastolic blood pressure*								
	Mean (mm Hg)	74 (12)	75 (12)	74 (12)	74 (11)	74 (12)	77 (12)	73 (11)
	Missing data	5195 (6%)	2716 (6%)	2479 (5%)	922 (5%)	1057 (6%)	2601 (12%)	645 (3%)
BMI category*								
	Underweight	2193 (4%)	1541 (6%)	652 (2%)	389 (4%)	424 (4%)	329 (3%)	592 (4%)
	Normal	17 381 (31%)	8413 (33%)	8968 (29%)	3665 (35%)	2967 (29%)	3000 (31%)	4368 (30%)
	Overweight	18 786 (34%)	7060 (28%)	11 726 (38%)	3741 (35%)	3220 (31%)	3434 (36%)	4629 (32%)
	Obese	17 644 (32%)	8222 (33%)	9422 (31%)	2789 (26%)	3793 (37%)	2910 (30%)	4784 (33%)
	Missing data	37 070 (40%)	20 411 (45%)	16 659 (35%)	7787 (42%)	5866 (36%)	12270 (56%)	6431 (31%)
Smoking*								
	No	29 551 (41%)	17 603 (53%)	11 948 (31%)	6394 (46%)	4496 (34%)	5081 (41%)	7023 (41%)
	Ex-smoker	32 572 (45%)	11 604 (35%)	20 968 (54%)	6248 (45%)	5838 (45%)	5192 (42%)	7949 (47%)
	Yes	9596 (13%)	3929 (12%)	5667 (15%)	1146 (8%)	2755 (21%)	2031 (17%)	2065 (12%)
	Missing data	21 355 (23%)	12 511 (27%)	8844 (19%)	4583 (25%)	3181 (20%)	9639 (44%)	3767 (18%)
Comorbidities								
	Atrial fibrillation	36 950 (40%)	18 309 (40%)	18 641 (39%)	7711 (42%)	6044 (37%)	6990 (32%)	9460 (45%)
	Chronic kidney disease	22 762 (24%)	11 912 (26%)	10 850 (23%)	4325 (23%)	3956 (24%)	1363 (6%)	7542 (36%)
	Chronic obstructive pulmonary disease	17 896 (19%)	8199 (18%)	9697 (20%)	2670 (14%)	4343 (27%)	3782 (17%)	4494 (22%)
	Diabetes	20 531 (22%)	9363 (21%)	11 168 (23%)	3489 (19%)	4238 (26%)	3893 (18%)	5366 (26%)
	Dyslipidaemia	25 958 (28%)	11 516 (25%)	14 442 (30%)	5062 (28%)	4948 (30%)	3361 (15%)	8024 (39%)
	Hypertension	62 419 (67%)	32 117 (70%)	30 302 (64%)	12 230 (67%)	11 008 (68%)	11 940 (54%)	15 766 (76%)
	Ischaemic heart disease	45 584 (49%)	19 408 (42%)	26 176 (55%)	8745 (48%)	8317 (51%)	10 279 (47%)	10 341 (50%)
	Osteoarthritis	40 176 (43%)	23 040 (50%)	17 136 (36%)	7828 (43%)	7186 (44%)	7962 (36%)	10 277 (49%)
	Three or more comorbidities	73 610 (79%)	37 338 (82%)	36 272 (76%)	14 188 (77%)	13 236 (81%)	14 876 (68%)	18 040 (87%)

Data are mean (SD) or n (%). Socioeconomic status refers to Index of Multiple Deprivation 2015 quintile, with SES 1 referring to the most affluent and SES 5 to the most deprived socioeconomic quintile. Number of comorbidities refers to any of the 17 conditions investigated. BMI=body-mass index.

* Number and percentage of records with missing data are displayed for variables with missing entries. Category percentages refer to complete cases.

**Figure 1:**
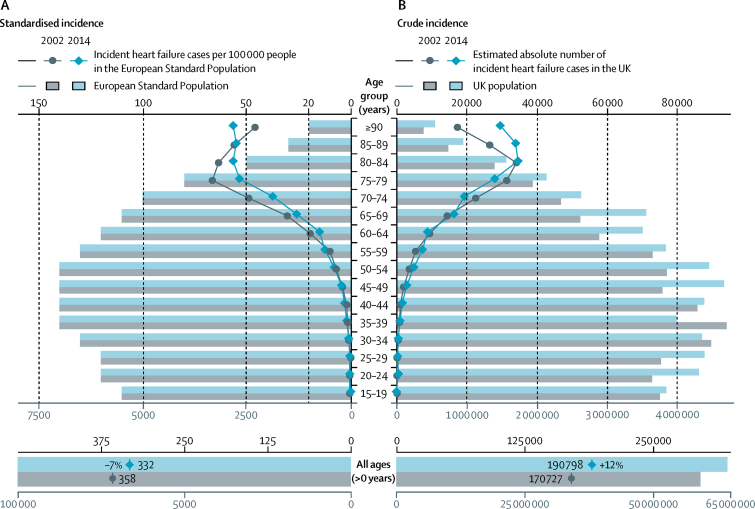
Overall and age-stratified heart failure incidence in 2002 versus 2014 (A) Number of cases of incident heart failure per 100 000 people in the European Standard Population. (B) Estimated absolute number of cases of incident heart failure in the UK population (based on census mid-year estimates).

In models standardised for age and sex, incidence of heart failure decreased by 7%, from 358 per 100 000 people in 2002 to 332 per 100 000 people in 2014 (adjusted incidence rate ratio [IRR] 0·93, 95% CI 0·91–0·94; figure 1A). This overall decline was consistent across most age groups. However, there was an increase in incidence in the very elderly (85 years or older), and in those younger than 55 years the rates were too low for a meaningful contribution to the overall burden. By contrast with the declining standardised incidence, crude incidence increased by 2%, from 288 per 100 000 people in 2002 to 295 per 100 000 people in 2014, and the estimated absolute number of yearly new diagnoses of heart failure increased by 12% from 170 727 in 2002 to 190 798 in 2014, due to population growth, especially in older age groups (figure 1B). For comparison, we aggregated the number of new cases of the four most common causes of cancer (lung, breast, bowel, and prostate) reported by Cancer Research UK in 2014,^25^ which totalled 189 136.

Prevalence of heart failure, standardised by age and sex, remained relatively stable during the study period, ranging from 1·5% in 2002 to 1·6% in 2014 (adjusted rate ratio 1·03, 95% CI 1·02–1·05). Nonetheless, the absolute number of people living with heart failure in the UK increased by 23% over the study period, from 750 127 in 2002 (1·3% of the total population) to 920 616 in 2014 (1·4% of the total population; appendix).

**Figure 2:**
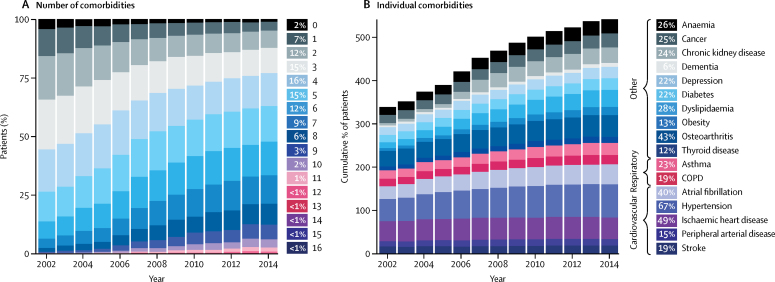
Temporal trends in comorbidities among patients diagnosed with incident heart failure, from 2002 to 2014 (A) Number of comorbidities, out of 17 major conditions, affecting patients with incident heart failure, over time. (B) Cumulative percentage of patients affected by individual comorbidities, over time. COPD=chronic obstructive pulmonary disease.

The number of comorbidities at or before diagnosis of incident heart failure was high (mean 4·5 [SD 2·4]) and increased over time, from 3·4 (SD 1·9) in 2002 to 5·4 (SD 2·5) in 2014 (difference adjusted for age, sex, and socioeconomic status 2·0, 95% CI 1·9–2·1; figure 2). Overall, 79% patients had three or more comorbidities, increasing from 68% in 2002 to 87% in 2014 (table). The most common comorbidities preceding a diagnosis of heart failure are shown in figure 2B.

Age-standardised incidence of heart failure was higher in men than in women (IRR 1·52, 95% CI 1·50–1·54), particularly in younger age groups, such as the 45–54 years age group (IRR 2·23, 95% CI 2·08–2·39). However, the total number of incident cases was only 9% higher in men, because of the greater number of women in the older age groups (appendix). Men were also younger at diagnosis than were women (mean age 74·0 years [SD 12·7] *vs* 79·4 years [11·8]; adjusted difference −5·51, 95% CI −5·67 to −5·35; table). In time-trend analyses, the rate of decline in age-standardised incidence was similar for men and women from 2002 to 2014. A similar pattern was seen in patients with prevalent heart failure; standardised prevalence was higher in men than in women (1·8% in men *vs* 1·2% in women; adjusted rate ratio (RR) 1·52, 95% CI 1·51–1·53).

At same age and sex, patients in the most deprived socioeconomic quintile were more likely to experience incident heart failure (IRR 1·61, 95% CI 1·58–1·64) than were affluent individuals. Socioeconomic inequalities were apparent across all age groups and both sexes but were more pronounced in younger age groups (IRR 2·56, 95% CI 2·30–2·85 in the 45–54 years age group *vs* 1·17, 95% CI 1·13–1·22 in the >85 years age group). A similar disparity was observed in prevalence standardised by age and sex (2·0% in most deprived *vs* 1·2% in least deprived; adjusted RR 1·57, 95% CI 1·55–1·58).

**Figure 3:**
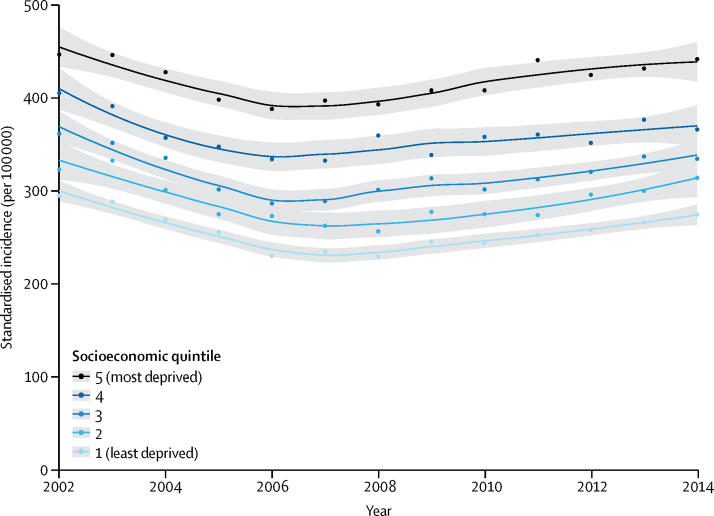
Temporal trends in heart failure incidence by socioeconomic status quintile (2002–14) Age and sex-standardised incidence per 100 000 people by year and socioeconomic quintile are presented with fitted local polynomial regression lines and 95% CIs in grey. Socioeconomic quintile refers to Index of Multiple Deprivation 2015 quintile.

**Figure 4:**
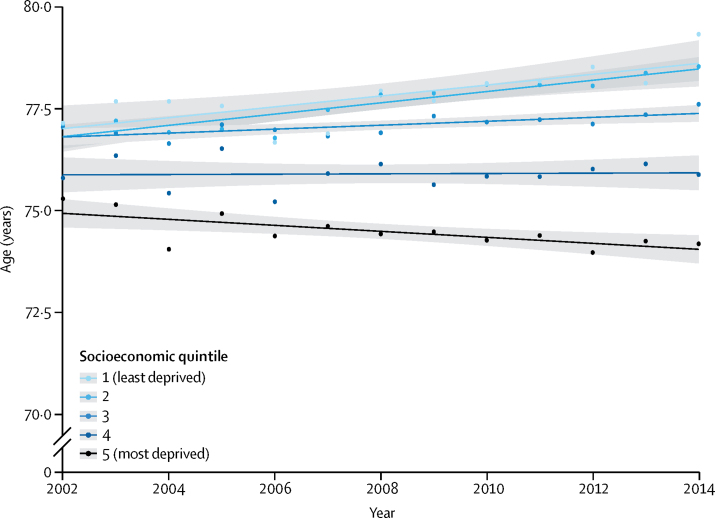
Temporal trends in age at diagnosis of incident heart failure by socioeconomic status quintile (2002–14) Mean age at incident heart failure diagnosis by year and socioeconomic quintile is presented with fitted linear regression lines and 95% CIs in grey. Socioeconomic quintile refers to Index of Multiple Deprivation 2015 quintile.

In stratified time-trend analyses, there was no evidence to suggest that the overall decline in incidence, standardised by age and sex, differed by socioeconomic quintiles (figure 3). However, age at which heart failure was diagnosed differed by socioeconomic status and this gap in age widened over time (figure 4). During the study period, patients from the most deprived socioeconomic quintile were about 3·5 years younger at diagnosis than those from the least deprived (mean age at diagnosis 74·5 years [SD 13·3] for most deprived *vs* 77·8 years [SD 12·1] for most affluent group; adjusted difference −3·51 years, 95% CI −3·77 to −3·25). From 2002 to 2014, mean age at diagnosis increased by 2·45 years (95% CI 1·58–3·32) among the most affluent, but tended to decrease amongst the most deprived (adjusted difference −0·44 years, 95% CI −1·50 to 0·61; figure 4). Socioeconomic inequalities were also visible in comorbidity rates, and the proportion of patients presenting with three or more comorbidities ranged from 81% for the most deprived to 77% for the least deprived group (table).

If incidence from the most affluent group could be achieved for all socioeconomic groups, we would expect 31 810 fewer heart failure cases annually in the UK, or an approximately 18% lower crude incidence.

Regional variation in heart failure incidence was wide, but attenuated over the study period (appendix).

## Discussion

This large-scale, representative, population-based study provides several insights into the burden of heart failure in the UK and its variation over time, by age, sex, region, and socioeconomic status.

The incidence rate of heart failure, standardised by age and sex, decreased modestly between 2002 and 2014, which contrasts with myocardial infarction, for which incidence has decreased by about a third over a similar time period in many countries.^26^ The comparatively small reduction in standardised incidence of heart failure might be due to higher survival among patients with post-myocardial infarction ventricular dysfunction as a result of improvements in medical treatment.^26^, ^27^, ^28^ Further insights into these trends come from our stratified analyses. Although the declines in incidence were largely consistent for both sexes, across all socioeconomic groups and regions the age-stratified analyses show some discordant trends. In the very elderly (>85 years), the standardised incidence appears to have increased, despite decreasing for myocardial infarction in the same age category.^26^, ^29^

The increase in incidence of heart failure in older people could result from less effective control of cardiovascular risk factors in those age groups. Alternative explanations include policy interventions for better diagnosis and management of patients in the community,^12^ and investments in specialist heart failure services that might have led to increased diagnosis rates, particularly among the elderly. Such national policy interventions and investments in services could also explain the nadir we noted in the number of new heart failure diagnoses in the years 2004–06, which coincides with the introduction of a national care monitoring programme, the Quality and Outcomes Framework. However, such modest short-term variations have also been noted in other long-term heart failure incidence studies,^3^, ^4^ and could be random variation.

Standardised trends are necessary to compare changes in disease burden over time and place, but to service payers, providers, and researchers, it is also important to know the absolute numbers of patients who require treatment. Despite a modest decline in standardised incidence, there has been a 12% increase in the total number of new cases of heart failure. This is substantial, and by comparison is now similar to the total number of new cases of breast, prostate, lung, and bowel cancer combined.^25^ The predominant reason for the rise in incidence is the increasing number of older people in the UK, a demographic change in many other countries. More specifically, the number of people aged 65–69 years has increased by 36%, representing the post-war baby-boom generation that is now reaching the age at which risk of heart failure increases.

The prevalence of heart failure has increased even more substantially than its incidence, possibly as a result of longer survival after heart failure diagnosis. Overall, despite a relatively stable standardised prevalence, we observed a 23% increase in the absolute number of people living with heart failure.

The number of comorbidities associated with heart failure was high and increased between 2002 and 2014 in parallel, with increasing age at onset. This suggests that in addition to the growing number of patients with heart failure, their clinical management is becoming more complex, further increasing the burden on health services and demand for specialist cardiology input in multidisciplinary care teams. The increasing number of comorbidities is likely to be influenced by several factors, such as population ageing, enhanced screening and diagnostics, physician awareness, and changes in risk factors. Beyond the number of comorbidities, the nature of comorbidities noted in our study reveals interesting patterns. For instance, non-cardiovascular comorbidities make a substantial contribution to multimorbidity in patients with heart failure and they have been doing so increasingly over time. Future appropriately designed studies could investigate this question further.

Achieving equity in access to essential health care has been a goal of many health-care systems and is now a specific target of the UN's Sustainable Development Goal 3. Previous studies^30^ have shown that in countries where the human development index (a composite measure of gross national income per capita, life expectancy, and education) is low, patients present with heart failure at a much younger age than in countries with a high human development index. Likewise, at the country level, socioeconomic deprivation is associated with an increased incidence of heart failure.^7^, ^31^ The results of our study build upon these findings by showing that low socioeconomic status is not only associated with higher standardised incidence of heart failure, but also an earlier disease onset and higher comorbidity rates than associated with high socioeconomic status. Our time-trend analyses further show that there has been no substantial convergence of the incidence rates by socioeconomic status and, in fact, the age gap between the most and least deprived groups widened by almost 2·5 years during the study period.

Our study was not designed to investigate the causal role of socioeconomic status or pathways through which it affects heart failure incidence. However, previous research has shown that the variability in heart failure incidence by socioeconomic gradient is likely to be explained by several biological, environmental, and behavioural risk factors, including, but not exclusively, known cardiovascular risk factors such as smoking, obesity, or blood pressure.^31^, ^32^ Thus, achieving equity in outcomes is likely to require additional population-level and individual-level interventions. The persistent and partly widening gaps in disparities in a health-care system with equality of access to health care also raises questions about how, when, and where more deprived patients seek care, and whether additional efforts are necessary to achieve equity in health-care use (as well as access).

A major strength of this study is the selection of a large representative cohort across primary and secondary care, with a sufficient number of cases in each sex, age, socioeconomic, and regional category to allow overall and subpopulation analyses. This population-based approach increases the generalisability of the findings compared with surveys that select partipants.^33^ Moreover, in view of the similarity of trends in cardiovascular disease and population ageing from the UK with other European countries, North America, and Australasia,^34^, ^35^ our findings are likely to be broadly applicable to much of the rest of the developed world.

One of the key limitations of our study was the incomplete clinical information contained in the available electronic health records. In particular, left ventricular ejection fraction values were not available, meaning that it was not possible to identify the type of heart failure. Research using electronic health records databases is also reliant on the accuracy of clinical coding input by physicians. The validity of clinical diagnoses recorded in CPRD has been investigated for a wide range of diseases, including heart failure^36^ and other common chronic conditions,^37^ with an average positive predictive value of 89%^38^ and 92% completeness, compared with national registry data.^39^ Moreover, national clinical audit programmes (particularly the Quality and Outcomes Framework and the National Heart Failure Audit) ensure stable quality of clinical coding practices and provide a solid support for the validity of recorded diagnoses. Indeed, these programmes report that approximately 90% of recorded heart failure diagnoses in England are referred for echocardiography, specialist assessment, or B-type natriuretic peptide measurement (appendix).^12^, ^14^

Our findings have implications for health-care resource planning and preventive strategies. The decline in standardised heart failure incidence, particularly in people aged 60–79 years, suggests that heart failure prevention has improved, probably because of a combination of environmental changes, public health measures, and improvements in clinical care. However, the increase in the absolute number of incident and prevalent cases of heart failure is placing an increasing burden on health care. The profile of patients with heart failure is diverse and evolving over time—with a trend towards older age and a substantial increase in the number of associated comorbidities—indicating that both prevention and management are becoming more complex. The observed disparities in heart failure incidence by sex, socioeconomic status, and region point to potential opportunities for more targeted and equitable prevention strategies.
