# Supplementary material for: Temporal trends and patterns in heart failure incidence: a population-based study of 4 million individuals
Source: Lancet. 2018 Feb 10;391(10120):572–80. doi: 10.1016/S0140-6736(17)32520-5 (PMC5814791; doi:10.1016/S0140-6736(17)32520-5)
Supplement: Supplementary appendix [file mmc1.pdf]

THE LANCET

Supplementary appendix

This appendix formed part of the original submission and has been peer reviewed. We post it as supplied by the authors.

Supplement to: Conrad N, Judge A, Tran J, et al. Temporal trends and patterns in heart failure incidence: a population-based study of 4 million individuals. *Lancet* 2017; published online Nov 21. http://dx.doi.org/10.1016/S0140-6736(17)32520-5.

Temporal trends and patterns in heart failure incidence: a population-based study of 4 million individuals

Supplementary Material

Text S1: Index of Multiple Deprivation 2
Text S2: Regional variation in heart failure incidence 3
Text S3: Validity of diagnoses recorded in electronic health record databases 4
Table S1: Selected studies reporting heart failure incidence in the general population. 5
Table S2: Clinical codes used to identify patients with heart failure. 7
Table S3: Clinical codes used to exclude first heart failure diagnoses not referring to an acute event. 10
Figure S1: Overall and age-stratified heart failure prevalence in 2002 versus 2014 12
Figure S2: Overall and age-stratified heart failure incidence for women and men. 13
Figure S3: Age-sex-standardised heart failure incidence in England by regions in 2002 and 2014 14

Text S1: Index of Multiple Deprivation

Index of Multiple Deprivation (IMD) scores are the most commonly used measure of socio-economic levels in the UK and have been widely used in healthcare research.[1–4] The overall index covers seven dimensions of deprivation including income, employment, education, health, crime, housing, living environment.[1,5] IMD is a broad measure that may further correlate with non-socio-economic factors including environmental, behavioural and biological factors. While social heterogeneity is inevitably present in small neighbourhoods, the small area boundaries remain fixed over time allowing consistent comparisons of temporal trends, and the small mean population improves the population homogeneity compared with other measures of neighbourhood deprivation.[1,5].

References

1. Department for Communities and Local Government (DCLG). The English Index of Multiple Deprivation 2015: Guidance [Internet]. 2015 [cited 2017 Feb 8]. Available from: https://www.gov.uk/government/statistics/english-indices-of-deprivation-2015
2. Newton JN, Briggs ADM, Murray CJL, et al. Changes in health in England, with analysis by English regions and areas of deprivation, 1990–2013: a systematic analysis for the Global Burden of Disease Study 2013. Lancet 2015;386(10010):2257–74.
3. Ramsay SE, Morris RW, Whincup PH, et al. The influence of neighbourhood-level socioeconomic deprivation on cardiovascular disease mortality in older age: longitudinal multilevel analyses from a cohort of older British men. J Epidemiol Community Health [Internet] 2015;69(12):1224–31. Available from: http://jech.bmj.com/content/early/2015/08/18/jech-2015-205542.full
4. Adams J, White M. Removing the health domain from the Index of Multiple Deprivation 2004--effect on measured inequalities in census measure of health. J Public Health (Bangkok) [Internet] 2006 [cited 2017 Jul 20];28(4):379–83. Available from: https://academic.oup.com/jpubhealth/article-lookup/doi/10.1093/pubmed/fdl061
5. Hawkins NM, Scholes S, Bajekal M, et al. Community care in England: reducing socioeconomic inequalities in heart failure. Circulation 2012;126(9):1050–7.

Text S2: Regional variation in heart failure incidence

Little is known about regional variation in heart failure incidence in England, and how it has evolved over time. In supplementary analyses, we investigated how incidence rates varied by region and time.

Region is recorded at practice level by Clinical Practice Research Datalink (CPRD), and reflects the former geographical division of the National Health Service in 10 Strategic Health Authorities in England. These correspond to administrative regions, except that the large South East England region is divided into two: South Central and South East Coast.[6]

We observed substantial regional variation in heart failure incidence rates even after adjusting for age, sex, and socio-economic differences. Age-sex-standardised incidence rates ranged from 268 per 100,000 patient-years in the 'South East Coast' region to 393 per 100,000 patient-years in the 'North West' region (incidence rate ratio (IRR) 1.35, [1.32,1.38]). However, these overall regional differences attenuated over the study period, so that IRR between the highest and lowest incidence regions decreased from 1.44 [1.31,1.58] in 2002 to 1.26 [1.15,1.38] in 2014. Regions with highest heart failure incidence rates were those located in the North of England (North West, East Midlands, North East, Yorkshire and the Humber, West Midlands) (**Figure S3**).

The English 'North-South' inequality in health has been widely reported,[7–9] with cardiovascular diseases as the largest factor behind health inequalities.[2,10] Previous studies have also shown inequalities to remain persistent after adjustment for socio-economic deprivation[9] and only partly explained by common cardiovascular risk factors such as systolic blood pressure, body mass index, or smoking[11]. Many other factors might plausibly explain the excess heart failure incidence in the north of England. These include concomitant medical conditions and their treatments (eg. respiratory diseases, depression, musculoskeletal affections, or infections) often linked to environmental (eg. working conditions or air pollution) and lifestyle (eg. diet or drug prescription habits) factors. Observed disparities offer potential opportunities to design more targeted and equitable prevention strategies, and suggest that prevention measures in England may need to prioritize northern regions to reduce inequalities. Further research is needed to understand the determinants of geographical disparities.

References

6. National Health Service. Strategic Health Authority Configurations [Internet]. 2006 [cited 2017 Feb 28]. Available from: http://www.nhshistory.net/MapofSHAsFeb09.pdf

7. Hacking JM, Muller S, Buchan IE. Trends in mortality from 1965 to 2008 across the English north-south divide: comparative observational study. BMJ 2011;342.

8. Law MR, Morris JK. Why is mortality higher in poorer areas and in more northern areas of England and Wales? J Epidemiol Community Heal 1998;(52):344–352.

9. Siegler V, Langford A, Johnson B. Regional differences in male mortality inequalities using the National Statistics Socio-Economic Classification, England and Wales, 2001–03 [Internet]. 2008 [cited 2017 Jul 21]. Available from: http://www.eurohex.eu/bibliography/pdf/Smith-HSQ-2008-2561717250/Smith-HSQ-2008.pdf#page=6

10. McFadden E, Luben R, Wareham N, Bingham S, Khaw K-T. Occupational social class, educational level, smoking and body mass index, and cause-specific mortality in men and women: a prospective study in the European Prospective Investigation of Cancer and Nutrition in Norfolk (EPIC-Norfolk) cohort. Eur J Epidemiol [Internet] 2008 [cited 2017 Jul 26];23(8):511–22. Available from: http://link.springer.com/10.1007/s10654-008-9267-x

11. Kumar R, Dalton AR. The English North-South divide: risk factors for cardiovascular disease accounting for cross-sectional socio-economic position. Perspect Public Health [Internet] 2014 [cited 2017 Jul 21];134(6):339–45. Available from: http://journals.sagepub.com/doi/10.1177/1757913913493236

Text S3: Validity of diagnoses recorded in electronic health record databases

Research using electronic health records databases is reliant on the accuracy of clinical coding input by physicians in primary care, as part of a consultation, or secondary care, as part of a hospital admission. The validity of diagnoses underlying our study has therefore been carefully assessed and was considered appropriate in light of the following arguments.

Independent validation studies. Three studies are of major importance: (i) a systematic review, published in 2010, reports 212 validation studies over a broad range of conditions with an average positive predictive value of 89%[12]; (ii) a study specifically investigating heart failure diagnoses, which despite it being conducted before the introduction of national care monitoring programmes reports a positive predictive value of 82%[13]; and (iii) a more recent study investigating the validity of chronic obstructive pulmonary disease (COPD), another major chronic condition managed in primary care, which reports an accuracy of 87% compared with specialist assessment.[14]

National care monitoring programmes. Two national clinical audit programmes (in particular the 'quality and outcomes framework' (QOF) introduced in 2004 for primary care, and the 'national heart failure audit' (NHFA) introduced in 2007 for secondary care) ensure a stable quality of clinical coding practices and provide a solid support for the validity of recorded diagnoses. Indeed, these report that approximately 90% of recorded heart failure diagnoses in England are referred for echocardiography, specialist assessment, or B-type natriuretic peptide (BNP) measurement.[15,16]

Clinical guidelines. Guidelines for the diagnosis and management of heart failure from the National Institute for Clinical Excellence (NICE)[17,18] provide additional consistency over the study period. Indeed, guidelines are largely consistent in regard to heart failure diagnostic criteria and recommended investigations. One important change is the availability of natriuretic peptides testing and the variability in assay accuracy; these are however mainly used to exclude suspected cases, as opposed to confirming diagnoses, and therefore unlikely to impact disease incidence rates.[17,18]

Sensitivity analyses. Finally, to confirm the validity of heart failure cases included in our cohort, we performed the following sensitivity analyses. (a) case identification restricted to diagnostic codes included in national care monitoring programmes. While for our main analysis we intentionally expanded the diagnostic codes from the national audit programmes list with additional codes indicating a heart failure diagnosis, so as to ensure completeness; sensitivity analyses, restricting diagnostic codes to those used in the national audit programmes, found that 97% of patients in our cohort had a record heart failure used in the national clinical audit programmes, and led to no significant changes in the present results. (b) case identification restricted to diagnoses recorded in secondary care, or referred for specialist assessment or echocardiography. We further found that 92% of patients included in our cohort had a heart failure diagnosis recorded in secondary care, or either a referral to specialist cardiology service or echocardiography. While that proportion moderately increased over time, we found no significant change by sex or socio-economic status.

References:

12. Herrett E, Thomas SL, Schoonen WM, Smeeth L, Hall AJ. Validation and validity of diagnoses in the General Practice Research Database: a systematic review. Br J Clin Pharmacol 2010;69(1):4–14.
13. Johansson S, Wallander M-A, Ruigómez A, Rodríguez LAG. Incidence of newly diagnosed heart failure in UK general practice. Eur J Heart Fail 2001;3(2):225–31.
14. Quint JK, Müllerova H, DiSantostefano RL, et al. Validation of chronic obstructive pulmonary disease recording in the Clinical Practice Research Datalink (CPRD-GOLD). BMJ Open 2014;4(7):e005540.
15. Primary Care Domain ND. Quality and Outcomes Framework Report, England 2015-16 [Internet]. 2016 [cited 2017 Feb 27]. Available from: http://content.digital.nhs.uk/qof
16. Mitchell P, Marle D, Donkor A, et al. National Heart Failure Audit 2013-2014 [Internet]. 2015 [cited 2016 Oct 12]. Available from: https://www.ucl.ac.uk/nicor/audits/heartfailure/documents/annualreports/hfannual13-14-updated.pdf
17. National Institute for Clinical Excellence (NICE). Management of chronic heart failure in adults in primary and secondary care. Clinical Guideline 5. 2003.
18. National Institute for Health and Clinical Excellence (NICE). Chronic heart failure: management of chronic heart failure in adults in primary and secondary care (CG108) [Internet]. 2010. Available from: http://guidance.nice.org.uk/CG108/Guidance

Table S1: Selected studies reporting heart failure incidence in the general population.

Study	Country	Time Period	Population	Size	Design	Case Identification	Case Definition	Reported Incidence Rate/100,000 Person-years			Comments
								All (Trend)	Men (Trend)	Women (Trend)	
Levy 2002 (Framingham)[19]	USA	1950–1969	General Population	10,311 subjects	Prospective cohort	Population based screening, including physical examination, electrocardiography, and review of hospital records, physicians' records, and pathology reports.	Framingham heart failure diagnosis criteria	-	627	420	The study uses a prospectively collected population cohort and validated diagnoses. The number of cases is comparatively small.
		1970–1979							563 (←→)	311 (↓)	
		1980–1989							536 (←→)	298 (↓)	
		1990–1999							564 (←→)	327 (↓)	
Roger 2004 (Olmsted County)[20]	USA	1979-1984	General Population	4,537 HF cases	Prospective cohort	Outpatient and hospital discharge records	Clinical diagnosis codes, validated by Framingham criteria on a subset	-	360	284	The study uses a county-wide cohort and partly validated diagnoses. The number of cases is comparatively small.
		1985-1990							390 (←→)	292 (←→)	
		1991-1995							375 (←→)	260 (←→)	
		1996-2000							383 (←→)	315 (←→)	
Gerber 2015 (Olmsted County)[21]	USA	2000	General Population	2,762 HF cases	Prospective cohort	Outpatient and hospital discharge records	Clinical diagnosis codes, validated by Framingham criteria on a subset	316	Graphical (↓)	Graphical (↓)	The study uses a county-wide cohort, partly validated diagnoses, and distinguishes between HF-REF/HF-PEF. The number of cases is comparatively small.
		2010						219 (↓)			
Ezekowitz 2011 (Canada)[22]	Canada	2000	General Population	82,323 HF cases	Retrospective longitudinal cohort	GP, specialist, and hospital discharge records	Clinical diagnosis codes	538	-	-	The study focuses on incidence trends by place of diagnosis (e.g. emergency department vs. outpatient). Incidence rates are not stratified by sex or age-group.
		2006						403 (↓)			
Zarrinkoub 2013 (Sweden)[23]	Sweden	2006	General Population	2,056,173 subjects	Retrospective longitudinal cohort	GP, specialist, and hospital discharge records	Clinical diagnosis codes	-	390	360	The study presents incidence rates and trends by sex and age group in a large general population cohort. Considerations for changes in the denominator size over time and their impact on incidence rates are not presented.
		2010							290 (↓)	290 (↓)	

Study	Country	Time Period	Population	Size	Design	Case Identification	Case Definition	Reported Incidence Rate/100,000 Person-years			Comments
								All (Trend)	Men (Trend)	Women (Trend)	
Hawkins 2012 (CPRD 2007)[5]	UK	1999<br>2007	General Population	12,412 HF cases<br>13,330 HF cases	Retrospective longitudinal cohort	GP records	Clinical diagnosis codes	200<br>60 (↓)	-	-	The study presents stratification by socio-economic quintiles and incidence rates are standardised to the European Standard Population. Cases are identified based on primary, but not secondary, care records.
Cowie 1999 (Hillingdon)[24]	UK	1996	General Population > 25y	101,885 subjects	Prospective cohort	GP and hospital referrals	Clinical assessment, electrocardiography, chest radiography and transthoracic echocardiography	130 (○)	140 (○)	120 (○)	One of the first community-based heart failure incidence studies. The number of cases is comparatively small. This study does not report temporal trends within the cohort.
Murphy 2004 (Scotland)[25]	UK (Scotland)	1999-2000	General Population	307,741 subjects	Cross-sectional cohort	GP records	Clinical diagnosis codes associated with a ''first'' modifier	200 (○)	180 (○)	220 (○)	Cases are identified based on primary, but not secondary, care records. Age-standardised rates or temporal trends are not reported.
Gomez-Soto 2011 (Spain)[26]	Spain	2000<br>2007	General Population >=14	267,231 subjects	Prospective cohort	Diagnosis by GP or hospital admission	Framingham criteria	296<br>390 (↑)	306<br>400 (↑)	286<br>380 (↑)	The study reports crude rates. Age-standardised rates are not reported.

Abbreviations: Heart Failure (HF), General Practice (GP), United Kingdom (UK), United States of America (USA), European Society of Cardiology (ESC).
Trends: ←→ indicates stable trend, ↓ indicates declining trend, and ○indicates trends are not reported

References:

19. Levy D, Kenchaiah S, Larson MG, et al. Long-Term Trends in the Incidence of and Survival with Heart Failure. N Engl J Med 2002;347(18):1397–402.
20. Roger VL, Weston SA, Redfield MM, et al. Trends in heart failure incidence and survival in a community-based population. JAMA 2004;292(3):344–50.
21. Gerber Y, Weston S a, Redfield MM, et al. A contemporary appraisal of the heart failure epidemic in olmsted county, Minnesota, 2000 to 2010. JAMA Intern Med 2015;175(6):996–1004.
22. Ezekowitz JA, Kaul P, Bakal JA, Quan H, McAlister FA. Trends in heart failure care: has the incident diagnosis of heart failure shifted from the hospital to the emergency department and outpatient clinics? Eur J Heart Fail 2011;13(2):142–7.
23. Zarrinkoub R, Wettermark B, Wändell P, et al. The epidemiology of heart failure, based on data for 2.1 million inhabitants in Sweden. Eur J Heart Fail 2013;15(9):995–1002.
24. Cowie MR, Wood DA, Coats AJS, et al. Incidence and aetiology of heart failure; a population-based study. Eur Heart J 1999;20(6).
25. Murphy NF, Simpson CR, McAlister FA, et al. National survey of the prevalence, incidence, primary care burden, and treatment of heart failure in Scotland. Heart 2004;90(10):1129–36.
26. Gomez-Soto FM, Andrey JL, Garcia-Egido AA, et al. Incidence and mortality of heart failure: A community-based study. Int J Cardiol 2011;151(1):40–5.

Table S2: Clinical codes used to identify patients with heart failure.

A. ICD-10 codes used to identify patients with heart failure in hospital discharge records.

Code Type	Code	Description	# Incident Cases (%)	HF Type	Comment
ICD	I50.0	Congestive heart failure	22177 (23.83%)	HF-UNS	NHFA
ICD	I50.1	Left ventricular failure	20555 (22.08%)	HF-UNS	NHFA
ICD	I50.9	Heart failure, unspecified	7585 (8.15%)	HF-UNS	NHFA
ICD	I42.0	Dilated cardiomyopathy (Congestive cardiomyopathy)	657 (0.71%)	HF-REF	NHFA
ICD	I42.9	Cardiomyopathy, unspecified (Cardiomyopathy (primary)(secondary) NOS)	562 (0.6%)	HF-UNS	NHFA
ICD	I11.0	Hypertensive heart disease with (congestive) heart failure	430 (0.46%)	HF-UNS	NHFA
ICD	I25.5	Ischaemic cardiomyopathy	327 (0.35%)	HF-REF	NHFA
ICD	I13.2	Hypertensive heart and renal disease with both (congestive) heart failure and renal failure	52 (0.06%)	HF-UNS	
ICD	I13.0	Hypertensive heart and renal disease with (congestive) heart failure	25 (0.03%)	HF-UNS	
**Total Incident Cases**			**52,370**		

B. Read codes used to identify patients with heart failure in general practice records.

Code Type	Medcode	Read Code	Description	# Incident Cases (%)	HF Type	Comment
READ	884	G581.00	Left ventricular failure	9353 (10.05%)	HF-UNS	QOF HF
READ	2062	G58..00	Heart failure	7998 (8.59%)	HF-UNS	QOF HF
READ	2906	G580.11	Congestive cardiac failure	6462 (6.94%)	HF-UNS	QOF HF
READ	398	G580.00	Congestive heart failure	4874 (5.24%)	HF-UNS	QOF HF
READ	8966	G5yy900	Left Ventricular Systolic Dysfunction	1751 (1.88%)	HF-REF	QOF LVSD
READ	3204	G55..00	Cardiomyopathy	1284 (1.38%)	HF-UNS	
READ	11284	585f.00	Echocardiogram shows left ventircular systolic dysfunction	1139 (1.22%)	HF-REF	QOF LVSD
READ	1223	G58..11	Cardiac failure	1088 (1.17%)	HF-UNS	QOF HF
READ	5942	G581.13	Impaired left ventricular function	919 (0.99%)	HF-REF	QOF HF
READ	7251	33BA.00	Impaired Left Ventricular Function	852 (0.92%)	HF-REF	
READ	9913	1O1..00	Heart failure confirmed	746 (0.8%)	HF-UNS	

Code Type	Medcode	Read Code	Description	# Incident Cases (%)	HF Type	Comment
READ	12550	G5yyA00	Left Ventricular Diastolic dysfunction	471 (0.51%)	HF-UNS	
READ	8010	G551.00	Hypertrophic obstructive cardiomyopathy	398 (0.43%)	HF-REF	
READ	5695	G41z.11	Chronic cor pulmonale	390 (0.42%)	HF-UNS	
READ	4024	G58z.00	Heart failure NOS	304 (0.33%)	HF-UNS	QOF HF
READ	11351	585g.00	Echo shows LVDD	291 (0.31%)	HF-REF	
READ	7535	G554400	Primary dilated cardiomyopathy	229 (0.25%)	HF-REF	
READ	13189	662g.00	New York Heart Association classification - class II	180 (0.19%)	HF-UNS	QOF HF
READ	5255	G581000	Acute left ventricular failure	170 (0.18%)	HF-UNS	QOF HF
READ	10079	G580.12	Right heart failure	162 (0.17%)	HF-UNS	QOF HF
READ	3499	G554300	Hypertrophic non-obstructive cardiomyopathy	152 (0.16%)	HF-REF	
READ	16383	1O1..00	Heart failure confirmed	148 (0.16%)	HF-UNS	
READ	9524	G580.14	Biventricular failure	120 (0.13%)	HF-UNS	QOF HF
READ	7320	G343.00	Ischaemic cardiomyopathy	113 (0.12%)	HF-REF	
READ	17278	G58z.12	Cardiac failure NOS	100 (0.11%)	HF-UNS	QOF HF
READ	19066	662h.00	New York Heart Association classification - class III	98 (0.11%)	HF-UNS	QOF HF
READ	27964	G582.00	Acute heart failure	88 (0.09%)	HF-UNS	QOF HF
READ	22993	G55z.00	Cardiomyopathy NOS	79 (0.08%)	HF-UNS	
READ	107397	G5yyD00	Left ventricular cardiac dysfunction	79 (0.08%)	HF-REF	QOF LVSD
READ	18853	662f.00	NYHA class f - i	79 (0.08%)	HF-UNS	QOF HF
READ	101138	G583.00	Heart failure with normal ejection fraction	71 (0.08%)	HF-UNS	
READ	32671	G580100	Chronic congestive heart failure	65 (0.07%)	HF-UNS	QOF HF
READ	4915	G555.00	Alcoholic cardiomyopathy	65 (0.07%)	HF-REF	
READ	10154	G580.13	Right ventricular failure	60 (0.06%)	HF-UNS	QOF HF
READ	9402	G55y.11	Secondary dilated cardiomyopathy	55 (0.06%)	HF-REF	
READ	27884	G580200	Decompensated cardiac failure	52 (0.06%)	HF-UNS	QOF HF
READ	32898	8H2S.00	Admit heart failure emergency	26 (0.03%)	HF-UNS	
READ	21852	G554200	Familial cardiomyopathy	20 (0.02%)	HF-UNS	
READ	106897	G583.12	Heart failure with preserved ejection fraction	18 (0.02%)	HF-UNS	QOF HF
READ	104275	G584.00	Right ventricular failure	17 (0.02%)	HF-UNS	QOF HF

Code Type	Medcode	Read Code	Description	# Incident Cases (%)	HF Type	Comment
READ	5141	G554000	Congestive cardiomyopathy	16 (0.02%)	HF-UNS	
READ	11424	G580300	Compensated cardiac failure	16 (0.02%)	HF-UNS	QOF HF
READ	97780	G559.00	Arrhythmogenic right ventricular cardiomyopathy	16 (0.02%)	HF-REF	
READ	101137	G583.11	HFNEF - heart failure with normal ejection fraction	15 (0.02%)	HF-UNS	QOF HF
READ	106008	8CMW800	Heart failure clinical pathway	13 (0.01%)	HF-UNS	
READ	94870	G580400	Congestive heart failure due to valvular disease	12 (0.01%)	HF-UNS	QOF HF
READ	27683	G558100	Cardiomyopathy in myotonic dystrophy	9 (0.01%)	HF-UNS	
READ	70648	Gyu5M00	[X]Other hypertrophic cardiomyopathy	8 (0.01%)	HF-REF	
READ	22262	G1yz100	Rheumatic left ventricular failure	7 (0.01%)	HF-UNS	QOF HF
READ	51214	662i.00	New York Heart Association classification - class IV	7 (0.01%)	HF-UNS	QOF HF
READ	106198	661M500	Heart failure self-management plan agreed	7 (0.01%)	HF-UNS	
READ	103732	8CMK.00	Has heart failure management plan	4 (0%)	HF-UNS	
READ	62718	G21z100	Hypertensive heart disease NOS with CCF	2 (0%)	HF-UNS	
READ	52127	G211100	Benign hypertensive heart disease with CCF	2 (0%)	HF-UNS	
READ	21837	G232.00	Hypertensive heart&renal dis wth (congestive) heart failure	2 (0%)	HF-UNS	
READ	105542	8CeC.00	Preferred place of care for next exacerbation heart failure	2 (0%)	HF-UNS	
**Total Incident Cases**				**40,704**		

Abbreviations: 'HF-REF' refers to heart failure with reduced ejection fraction (highlighted in blue). 'HF-UNS' refers to heart failure with unspecified ejection fraction. 'NHFA' refers to codes used by the National Heart Failure Audit to identify patients with a heart failure diagnosis from hospital discharge records. 'QOF HF' refers to codes used by the 2014 'quality and outcomes framework' (QOF) to identify patients with a heart failure diagnosis from general practice records. 'QOF LVSD' refers to codes used by the 2014 QOF to identify patients with a left ventricular systolic dysfunction diagnosis from general practice records.

Table S3: Clinical codes used to exclude first heart failure diagnoses not referring to an acute event.

Code Type	Medcode	Readcode	Description
READ	95021	9N4s.00	Did not attend practice nurse heart failure clinic
READ	24503	8B29.00	Cardiac failure therapy
READ	95835	679X.00	Heart failure education
READ	26115	8HHb.00	Referral to heart failure nurse
READ	5155	23E1.00	O/E - pulmonary oedema
READ	90935	9hH..00	Exception reporting: heart failure quality indicators
READ	30749	9hH0.00	Excepted heart failure quality indicators: Patient unsuitabl
READ	34213	9h1..00	Exception reporting: LVD quality indicators
READ	11613	9h11.00	Excepted from LVD quality indicators: Patient unsuitable
READ	28649	9h12.00	Excepted from LVD quality indicators: Informed dissent
READ	15058	14A6.00	H/O: heart failure
READ	46912	14AM.00	H/O: Heart failure in last year
READ	83502	662p.00	Heart failure 6 month review
READ	12366	662T.00	Congestive heart failure monitoring
READ	30779	662W.00	Heart failure annual review
READ	32945	8CL3.00	Heart failure care plan discussed with patient
READ	17851	8HBE.00	Heart failure follow-up
READ	70619	8HHz.00	Referral to heart failure exercise programme
READ	71235	8Hk0.00	Referred to heart failure education group
READ	64062	9hH1.00	Excepted heart failure quality indicators: Informed dissent
READ	32911	9Or..00	Heart failure monitoring administration
READ	19380	9Or0.00	Heart failure review completed
READ	90193	9Or1.00	Heart failure monitoring telephone invite
READ	90192	9Or2.00	Heart failure monitoring verbal invite
READ	72965	9Or3.00	Heart failure monitoring first letter
READ	72386	9Or4.00	Heart failure monitoring second letter
READ	89650	9Or5.00	Heart failure monitoring third letter
READ	18793	9On..00	Left ventricular dysfunction monitoring administration

Code Type	Medcode	Readcode	Description
READ	60710	9On0.00	Left ventricular dysfunction monitoring first letter
READ	60721	9On1.00	Left ventricular dysfunction monitoring second letter
READ	72341	9On2.00	Left ventricular dysfunction monitoring third letter
READ	92305	9On3.00	Left ventricular dysfunction monitoring verbal invite
READ	96484	9On4.00	Left ventricular dysfunction monitoring telephone invite
READ	100784	2126400	Heart Failure Resolved
READ	102585	8HgD.00	Discharge from heart failure nurse service
READ	106680	8HTL000	Referral to rapid access heart failure clinic
READ	106836	8IB8.00	Referral to heart failure exercise programme not indicated
READ	106894	8IE1.00	Referral to heart failure exercise programme declined
READ	107981	8IE0.00	Referral to heart failure education group declined
READ	42999	12CR.00	FH: Hypertrophic obstructive cardiomyopathy

Figure S1: Overall and age-stratified heart failure prevalence in 2002 versus 2014.

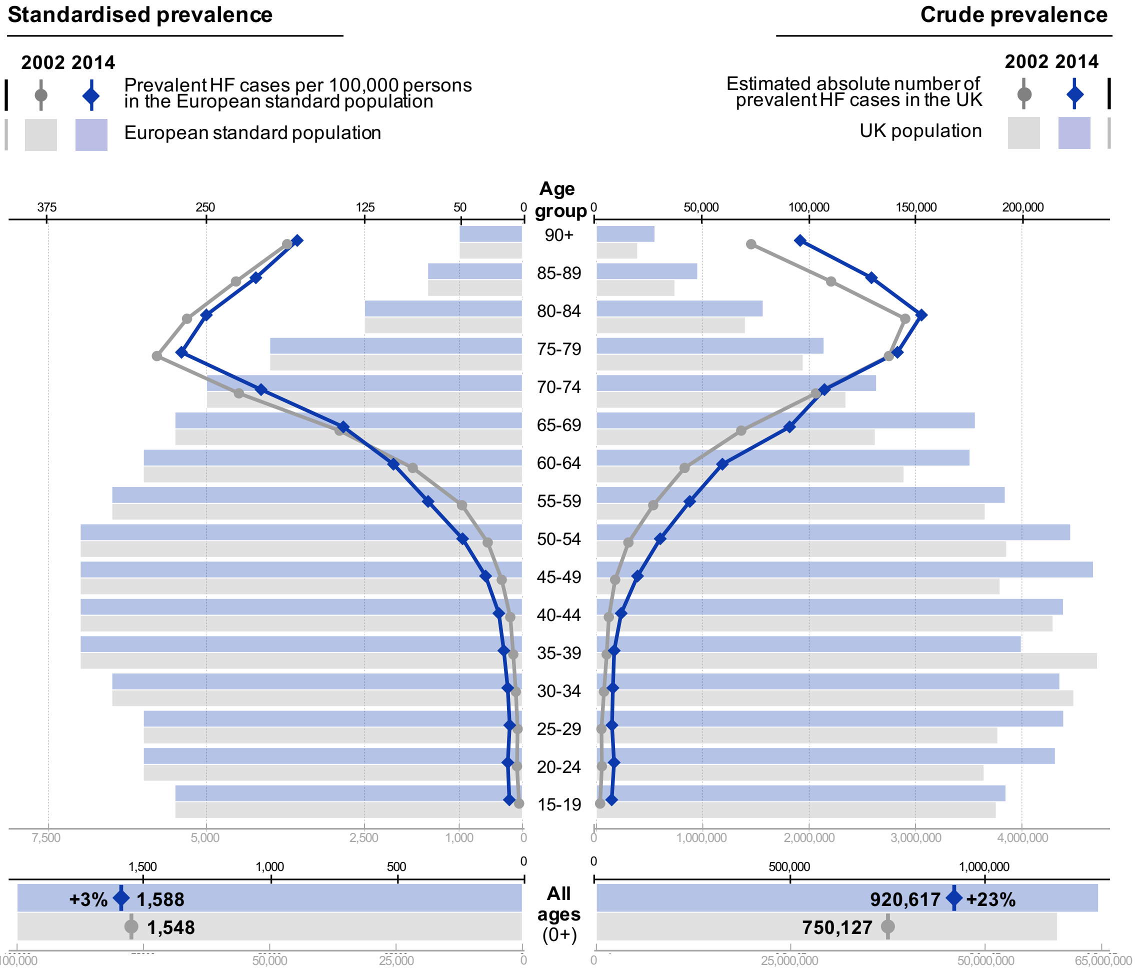

Standardised heart failure (HF) prevalence (left panel) presents cases in 100,000 persons from the European standard population. Crude prevalence *(right panel) presents estimated number of cases in the United Kingdom (UK) population (census mid-year estimates) in 2002 and 2014.*

Figure S2: Overall and age-stratified heart failure incidence for women and men.

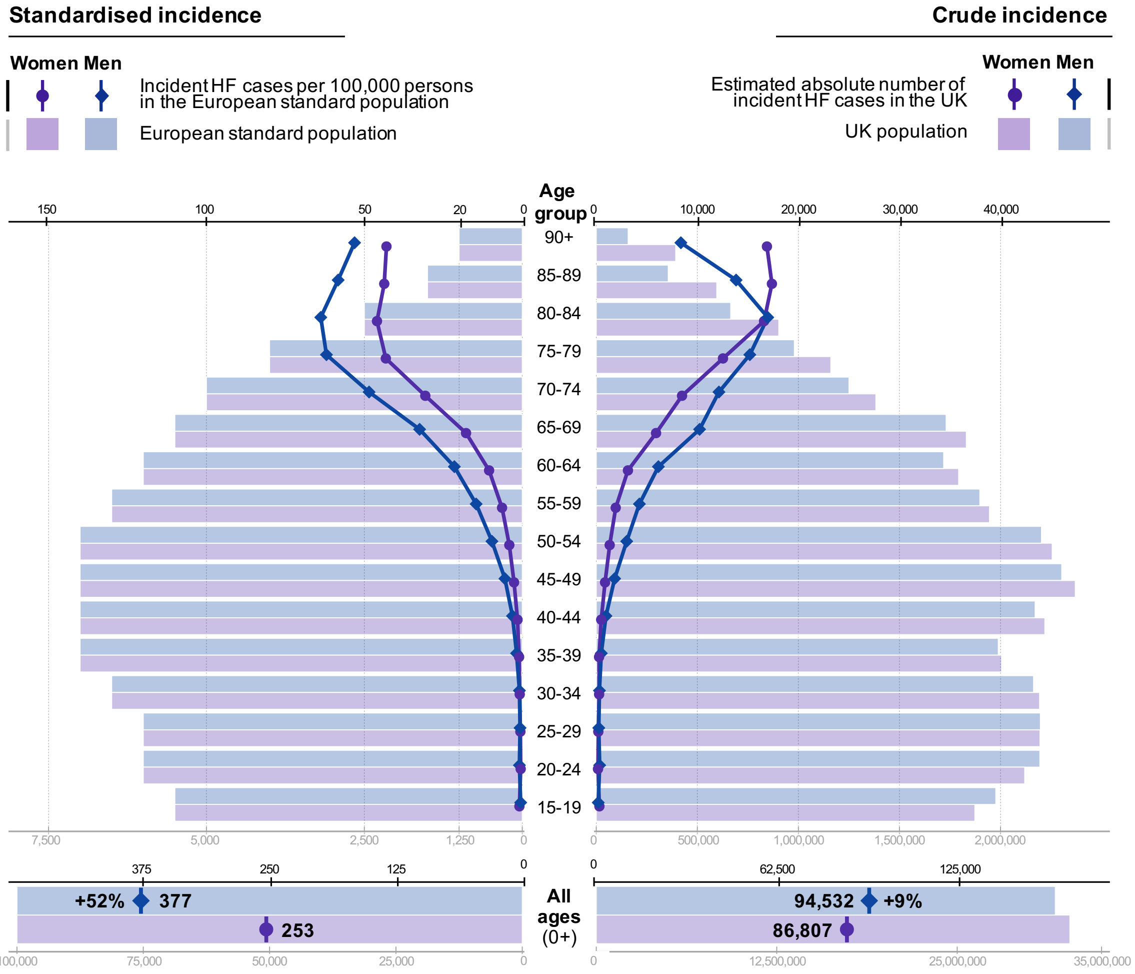

Standardised heart failure (HF) incidence (left panel) presents cases in 100,000 persons from the European standard population. Crude incidence (right panel) presents estimated absolute number of cases in the United Kingdom (UK) population (2014 census mid-year estimates). Incidence rates were calculated over all years from 2002 to 2014.

Figure S3: Age-sex-standardised heart failure incidence in England by regions in 2002 and 2014

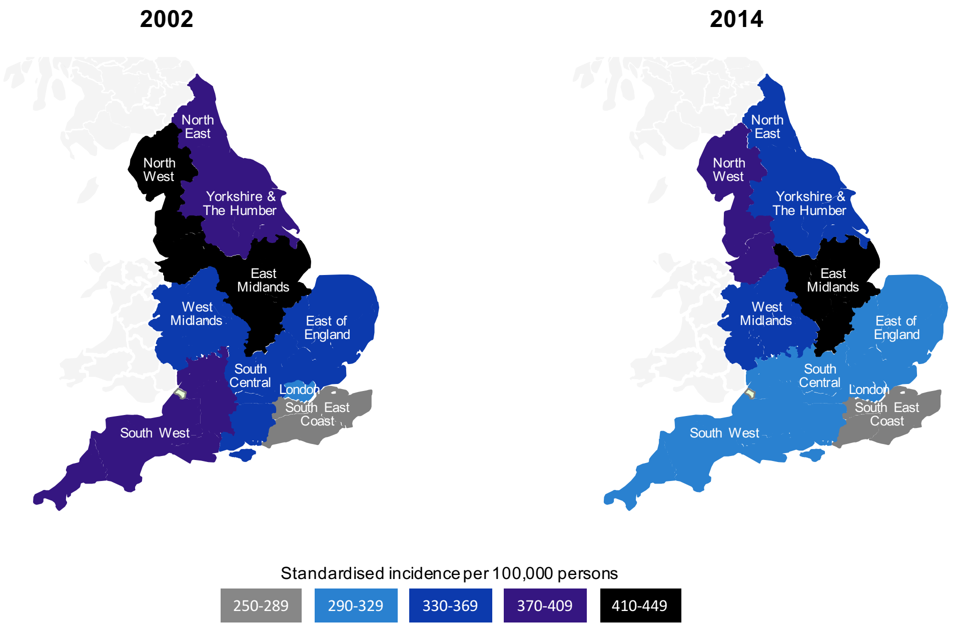

Incidence rates are standardised to the European standard population. Geographical division refers to the information provided by Clinical Practice Research Datalink (CPRD).
